# Supplementary material for: The impact of evolving maternal antiretroviral therapy guidelines on vertical transmission of HIV in the Western Cape, South Africa
Source: PLoS One. 2025 Sep 11;20(9):e0328612. doi: 10.1371/journal.pone.0328612 (PMC12425232; doi:10.1371/journal.pone.0328612)
Supplement: S1 Table — (DOCX) [file pone.0328612.s001.docx]

## **Supplementary Table**

**Table S1:Sensitivity analysis excluding child deaths: Comparison of different models for factors associated with vertical transmission of HIV based on routine data**

|  | **Policy periods 1 - 3** | | | | | | **Policy period 3** | | | |
| --- | --- | --- | --- | --- | --- | --- | --- | --- | --- | --- |
| **Variable†** |  |  | **Model 1** | | **Model 2** | | **Model 3** | | **Model 4** | |
|  | **OR** | **(95% CI)** | **aOR** | **(95% CI)** | **aOR** | **(95% CI)** | **aOR** | **(95% CI)** | **aOR** | **(95% CI)** |
| **Maternal ART Policy period** |  |  |  |  |  |  |  |  |  |  |
| Policy period 1 | 1.32 | 0.96-1.81 | 1.29 | 0.93-1.78 | 1.05 | 0.73-1.50 |  |  |  |  |
| Policy period 2 | 1.00 |  | 1.00 |  | 1.00 |  |  |  |  |  |
| Policy period 3 | **0.60** | 0.55 -0.65 | **0.67** | 0.61-0.73 | **1.14** | 1.02-1.27 |  |  |  |  |
|  |  |  |  |  |  |  |  |  |  |  |
| **Maternal age category** |  |  |  |  |  |  |  |  |  |  |
| 15 - 19 | **1.45** | 1.29-1.63 | **1.48** | 1.32-1.66 | **1.69** | 1.48-1.94 | **1.38** | 1.17-1.62 | **1.39** | 1.19-1.64 |
| 20-24 | **1.24** | 1.14-1.34 | **1.28** | 1.19-1.39 | **1.30** | 1.20-1.42 | **1.16** | 1.05-1.28 | **1.18** | 1.06-1.30 |
| 25-39 | 1.00 |  | 1.00 |  | 1.00 |  | 1.00 |  | 1.00 |  |
| >39 | 0.81 | 0.66-1.00 | 0.80 | 0.65-0.99 | **0.78** | 0.62-0.98 | 0.84 | 0.65-1.09 | 0.84 | 0.65-1.09 |
|  |  |  |  |  |  |  |  |  |  |  |
| **Electronic evidence of prior pregnancy** |  |  |  |  |  |  |  |  |  |  |
| 0 | 1.00 |  |  |  |  |  |  |  |  |  |
| 1 | **0.91** | 0.84-0.98 |  |  |  |  |  |  |  |  |
| 2 | **0.84** | 0.75-0.94 |  |  |  |  |  |  |  |  |
| 3 | 0.92 | 0.77-1.12 |  |  |  |  |  |  |  |  |
| ≥4 | 0.94 | 0.67-1.31 |  |  |  |  |  |  |  |  |
|  |  |  |  |  |  |  |  |  |  |  |
| **Maternal ART** |  |  |  |  |  |  |  |  |  |  |
| ART prior to pregnancy | 1.00 |  |  |  | 1.00 |  |  |  |  |  |
| Antenatal ART | **1.24** | 1.14-1.37 |  |  | **1.31** | 1.20-1.45 |  |  |  |  |
| No antenatal ART | **6.36** | 5.83-6.94 |  |  | **4.77** | 4.34-5.24 |  |  |  |  |
|  |  |  |  |  |  |  |  |  |  |  |
| **No recorded antenatal visits** | **1.71** | 1.59-1.84 | **1.57** | 1.46-1.69 | **1.35** | 1.24-1.46 | **1.12** | 1.02-1.24 | **1.13** | 1.02-1.24 |
| **Metropolitan district** | **0.75** | 0.69-0.80 | **0.84** | 0.78-0.91 | 0.94 | 0.87-1.02 | 0.99 | 0.91-1.09 | 0.99 | 0.90-1.09 |
| **History of TB** | **2.13** | 1.98-2.29 | **2.10** | 1.96-2.26 | **2.17** | 2.01-2.35 | **1.86** | 1.70-2.04 | **1.80** | 1.65-1.98 |
|  |  |  |  |  |  |  |  |  |  |  |
| **Viral Load and ART status ‡** |  |  |  |  |  |  |  |  |  |  |
| Suppressed, on ART before/during pregnancy | 1.00 |  |  |  |  |  | 1.00 |  | 1.00 |  |
| Viraemic, on ART before/during pregnancy | **5.11** | 4.59-5.68 |  |  |  |  | **4.68** | 4.20-5.22 | **4.50** | 4.03-5.02 |
| VL unavailable, on ART before/during pregnancy | **4.01** | 3.54-4.55 |  |  |  |  | **3.86** | 3.40-4.38 | **3.82** | 3.35-4.35 |
| VL unavailable, no antenatal ART record | **15.43** | 13.82-17.21 |  |  |  |  | **14.26** | 12.72-16.00 | **14.12** | 12.55-15.89 |
|  |  |  |  |  |  |  |  |  |  |  |
| **Last CD 4 before booking ‡** |  |  |  |  |  |  |  |  |  |  |
| CD < 200 | 1.00 |  |  |  |  |  |  |  | 1.00 |  |
| CD4 200 – 350 | **0.63** | 0.49-0.80 |  |  |  |  |  |  | 0.83 | 0.64-1.01 |
| CD4 >350 | **0.32** | 0.25-0.39 |  |  |  |  |  |  | **0.51** | 0.41-0.64 |
| CD4 result not available | **0.53** | 0.44-0.63 |  |  |  |  |  |  | **0.63** | 0.52-0.77 |

***†Outcome variable is child HIV status at 24 months.*** *Model 1 includes maternal ART policy period without adjustment for maternal ART, VL and CD4 count which are all impacted by the policy period. Model 2 includes both maternal ART policy period and maternal ART status. Model 3 includes VL status combined with ART status into a composite variable. Model 4 includes combined VL and ART status as well as CD4 status. Patients with VL status but no corroborating ART records were included in Models 1 and 2 but dropped from the models 3 and 4 (effect sizes remain unchanged).*

*‡Restricted to maternal ART policy period 3*
